# Effect of low-concentration carbohydrate on patient-centered quality of recovery in patients undergoing thyroidectomy: a prospective randomized trial

**DOI:** 10.1186/s12871-021-01323-8

**Published:** 2021-04-06

**Authors:** Shun Wang, Peng-fei Gao, Xiao Guo, Qi Xu, Yun-feng Zhang, Guo-qiang Wang, Jing-yan Lin

**Affiliations:** 1 Department of Anesthesiology, Affiliated Hospital of North Sichuan Medical College, Nanchong, 637000 Sichuan China; 2 Department of Anesthesiology, North Sichuan Medical College, Nanchong, 637000 Sichuan China

**Keywords:** Low-concentration carbohydrate, QoR-15 questionnaire, Insulin resistance, Thyroidectomy, Blood glucose

## Abstract

**Backround:**

At present, low-concentration carbohydrate is rarely used in minor trauma surgery, and its clinical efficacy is unknown. The aim of the study was to evaluate the effect of preoperative oral low-concentration carbohydrate on patient-centered quality of recovery in patients undergoing thyroidectomy using Quality of Recovery − 15 (QoR-15) questionnaire.

**Methods:**

One hundred twenty patients were randomized to oral intake of 300 ml carbohydrate solution (CH group) or 300 ml pure water (PW group) 2 h before surgery or fasting for 8 h before surgery (F group). The QoR-15 questionnaire was administered to compare the quality of recovery at 1d before surgery (T0), 24 h, 48 h, 72 h after surgery (T1, T2, T3), and perioperative blood glucose was recorded.

**Results:**

Compared to the F group, the QoR-15 scores were statistically higher in the CH and PW group at T1 (*P* < 0.05), and the enhancement of recovery quality reached the clinical significance at T1 in the CH group compared with the F group. Among the five dimensions of the QoR-15 questionnaire, physical comfort, physiological support and emotional dimension in the CH group were significantly better than the F group (*P* < 0.05) at T1. Besides, blood glucose of CH group was significantly lower than the PW group and F group at each time point after surgery.

**Conclusions:**

Low-concentration carbohydrate could decrease the incidence of postoperative hyperglycemia and improve the patient-centered quality of recovery on patients undergoing open thyroidectomy at the early stage postoperatively.

**Trial registration:**

ChiCTR1900024731. Date of registration: 25/07/2019.

## Background

Preoperative oral carbohydrate, guided by the theory of enhanced recovery after surgery (ERAS), has been used in more and more surgical operations to improve the quality of postoperative recovery through improving perioperative comfort, decreasing postoperative insulin resistance, reducing the incidence of postoperative nausea and vomiting (PONV) and shortening the postoperative hospital stay [1–3]. But the carbohydrate used in the clinic is almost all the high-concentration carbohydrate (≥ 12.5%), doctors don’t adjust the concentration of carbohydrate according to the patient’s condition in general. One of the main objectives of preoperative oral carbohydrate is to reduce postoperative insulin resistance. However, the degree of postoperative insulin resistance depends on the types of surgery, the postoperative insulin sensitivity of minor operations, such as laparoscopic cholecystectomy, is only 15 to 20% lower than that before surgery, while that of open cholecystectomy is about 75% lower than that before surgery [4, 5]. Perhaps it means we should adjust the concentration of carbohydrate according to the different types of surgery. So, it seems unreasonable that patients in all types of surgery were asked to take high-concentration carbohydrate solution. A meta-analysis pointed out that there was no significant difference between low- (< 12.5%) and high-concentration carbohydrates on the effect of postoperative recovery, such as length of postoperative stay, postoperative complication rate and so on, but there was little research on low-concentration carbohydrate, so the evidence of low-concentration carbohydrate about postoperative recovery quality is not convincing according to the current evidence [3]. Currently, the relative studies focus on the major operations and there is lack of evidence on minor surgeries. So, low-concentration carbohydrate may be sufficient to reduce insulin resistance and improve the quality of postoperative recovery for minor surgeries.

Quality of postoperative recovery is a comprehensive concept, which not only needs to be evaluated from the perspective of doctors but also fully considers the subjective feelings and emotions of patients. We chose an appropriate assessment tool: QoR-15 questionnaire [6],which developed in 2013 by Stark and his colleagues. It has been confirmed by many studies to full the requirements for appropriateness, reliability, validity, precision, acceptability, and feasibility in the assessment of postoperative recovery quality of adult general anesthesia [6–11]. Currently, Chinese version, which has the similar advantages as the English version, has also been developed [12]. QoR-15 is a patient-centered comprehensive questionnaire, which includes five aspects: physical comfort, psychological support, physical independence, emotional status and pain. We believe that QoR-15 can assess the effect of low-concentration carbohydrate on postoperative recovery accurately.

Taken together, we hypothesis that preoperative oral low-concentration carbohydrate may improve the patient-centered quality of postoperative recovery after minor surgeries. Therefore, this trial was designed to apply the QoR-15 questionnaire to evaluate the impact of preoperative oral low-concentration carbohydrate on the postoperative recovery quality after open thyroidectomy.

## Methods

### Ethics and registration

The Ethics Committee of the Affiliated Hospital of North Sichuan Medical College approved this prospective, double-blinded, randomized trial [2019ER(R)075–01], which registered at the Chinese Clinical Trials Registry [ChiCTR1900024731]. All methods were performed in accordance with the relevant guidelines and regulations, and all participants signed written informed consent.

### Patient inclusion and exclusion criteria

Patients ageing from 18 to 65 years, with an American Society of Anesthesiologists (ASA) physical status I–II, who was scheduled for elective open thyroidectomy at the Affiliated Hospital of North Sichuan Medical College were enrolled in the study. The exclusion criteria were as follows: (1) fasting glucose level ≥ 126 mg/dL (mg/dL = mmol/L × 18); (2) type 1 or 2 diabetes; (3) gastro-esophageal reflux disease; (4) pancreatic disease; (5) body mass index (BMI) ≥ 30 kg/m^2^; (6) refuse to participate in the study. Exit criteria were as follows: (1) cervical lymph node dissection was performed intraoperatively; (2) analgesics administration after surgery; (3) patients refused to follow-up.

### Randomization and blinding

The eligibility for inclusion was assessed in the ward 1d before surgery and the first QoR-15 score was performed. All enrolled patients were equally divided into three groups and administered with preoperative oral carbohydrate (CH group), pure water (PW group), and 8 h fasting before surgery (F group) by using a web-based random-number generator (available at www.random.org) on the day before surgery by the specific researcher who was only responsible for randomly grouping and implementing the intervention, the remaining researchers and the attending anesthesiologists were blinded to group assignment.

### Anesthesia and study protocol

Patients in the CH group were instructed to take the carbohydrate solution [4.8% carbohydrate, 88 kcal • 100 mL^− 1^, (lime taste), free of protein, fat, lactose and dietary fiber] orally 2 h before the planned time of operation (scheduled in advance). Patients in the PW group were instructed to drink pure water (vehicle used in the CH group) with the same timing and volume as those in the CH group. For patients in the F group, routine fasting procedure was implemented, in which patients were instructed not to take any fluid or food by mouth 8 h before surgery.

After entering the operating theatre, a rigorous preoperative ultrasound assessment was performed on every patient to evaluate the gastric volume (GV) in the supine position and right lateral decubitus. The cross-sectional area (CSA) of the gastric antrum, determining the gastric volume, was calculated according to the following formula using the anteroposterior (AP) and craniocaudal (CC) diameters, as described [13–19].

$$ \mathrm{CSA}\ \left({\mathrm{cm}}^2\right)=\pi \times \mathrm{AP}\times \mathrm{CC}/4 $$

$$ \mathrm{GV}\ \left(\mathrm{ml}\right)=27.0+14.6\times \mathrm{right}-\mathrm{lat}\ \mathrm{CSA}-1.28\times \mathrm{age}. $$

None of the patients received pre-anesthetic medications before surgery. Routine monitoring, including pulse oximetry, electrocardiogram, noninvasive arterial pressure, the bispectral index (BIS) were commenced upon arrival to the operating theatre. Anesthesia was induced using intravenous administration of midazolam 0.03–0.05 mg/kg, sufentanil 0.3–0.5 μg/kg, cis-atracurium 0.10–0.15 mg/kg and propofol 1.5–2.5 mg/kg. After endotracheal intubation, an anesthetic machine was used for controlled ventilation (VT 6–8 ml/kg and RR 12–16 times/min) to maintain an end-tidal carbon dioxide concentration between 30 and 45 mmHg. Continuous intravenous infusion of remifentanil and propofol, intermittent administration of cis-atracurium were administered for maintenance of anesthesia. About 30 min before end of the surgery, 10 μg of sufentanil was intravenously injected for analgesia and 4 mg of ondansetron was used for antiemetic prophylaxis. Remifentanil and propofol were ceased at end of the suture. After the operation, patients were extubated and sent to the postanesthesia care unit (PACU) after recovery of spontaneous breathing and consciousness. In all groups, the anesthetic depth was titrated to maintain a bispectral index (BIS) range between 40 and 60 through the target-controlled infusion (TCI) of propofol, and a target-controlled infusion of remifentanil was used to control the circulation within 20% of the pre-induction values. Under the appropriate depth of anesthesia, ephedrine (6 mg each time) was used when the noninvasive mean arterial pressure (MAP) was below 55 mmHg, urapidil hydrochloride (10 mg each time) was given when the noninvasive MAP was more than 110 mmHg. Atropine (0.5 mg each time) was injected when the heart rate (HR) was below 50 bpm, esmolol (10 mg each time) was used when the HR was more than 100 bpm. Perioperative pain was assessed by a numerical rating scale (NRS). Tramadol (100 mg) was given intravenously when the NRS scores was beyond 4. Postoperative nausea and vomiting (PONV) were treated with ondansetron (4 mg) intravenously.

### Outcomes

Outcomes were collected in operating rooms and hospital wards according to time points, the follow-up period began from 3 h after surgery and lasted until 3d.

Scores of QoR-15 was considered as the primary outcome. There were five dimensions as physical comfort (5 items), emotional state (4 items), physical independence (2 items), psychological support (2 items), and pain (2 items) included in QoR-15 questionnaire. Total scores of the QoR-15 ranges from 0 (the poorest quality of recovery) to 150 (the best quality of recovery). The QoR-15 questionnaire was administered at four time points: 1d before surgery (T0), 24 h, 48 h, 72 h after surgery (T1, T2, T3).

Secondary outcomes included the perioperative patient discomfort (including thirst, hunger, anxiety, evaluated at 1d before surgery, arrival in the operating theatre and 3 h, 24 h after surgery), gastric volume before surgery, vomiting and aspiration occurred during intubation and extubation, intraoperative vital signs, perioperative blood glucose (at admission, preoperatively, 1 h after incision, end of the surgery, 3 h after the surgery, every day after surgery at 4 PM for 3 consecutive days), PONV, time to gastrointestinal recovery, duration of the hospital stay after surgery. Besides, age, sex, ASA physical status, BMI, the consumption of anesthetics on the duration of anesthesia were also recorded.

### Sample size and statistical analysis

The sample size was estimated by the QoR-15 scores at 24 h after surgery, which measured through 10 patients per group. Considering a power of 90% with a type 1 error of 0.05, and a compliance rate of 80%, a total of 120 patients were enrolled in this trial (40 patients per group).

Analyses were performed by IBM SPSS Statistics 25.0. The hypothesis of normal distribution was test using the Kolmogorov-Smirnov test. Normally distributed data were reported as mean ± standard deviation (SD) and were analyzed using a one-way analysis of variance (ANOVA) or repeated measures analysis of variance. Non-normally distributed data were analyzed using the Kruskal-Wallis test and the Kruskal-Wallis one-way ANOVA were used for testing between groups. Categorical variables were compared using the chi-square test. A post hoc analysis with Bonferroni correction was performed. Statistically significant were considered as a *P*-value less than 0.05.

## Results

**Fig. 1:**
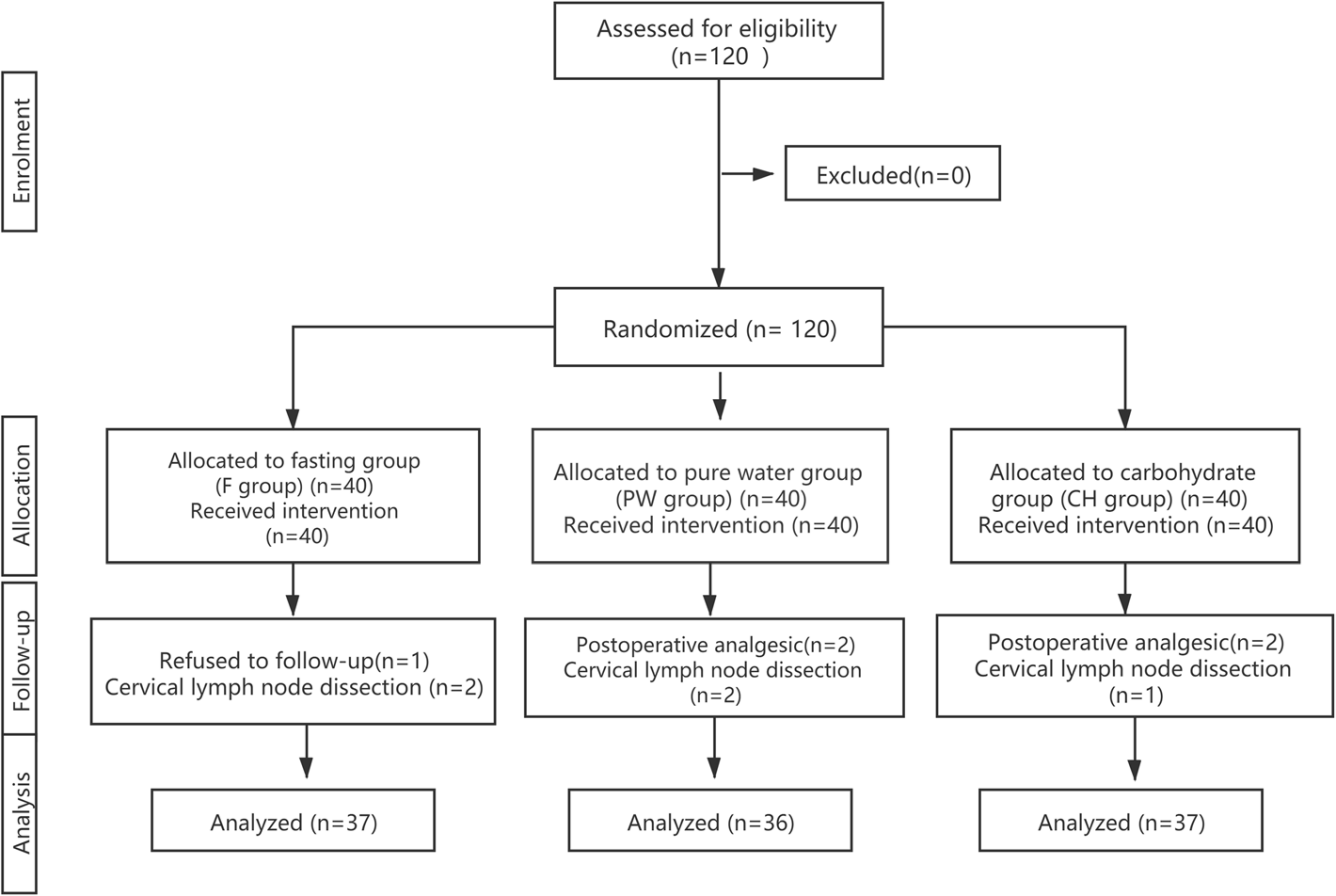
CONSORT flow diagram

From August 2019 to December 2019, 120 patients were screened for eligibility after applying the exclusion criteria and randomly assigned to three groups (CH, PW, and F group, *n* = 40). During this trial, 5 patients underwent cervical lymph node dissection, 4 patients were treated with analgesics after surgery, 1 patient refused to follow-up, therefore 10 patients were excluded from the study. As a result, data from a total of 110 patients were included for analysis (Fig. 1). The demographic characteristics exhibited no significant differences among the three groups (Table 1).

**Table 1:** Patients characteristics among groups

	F Group	PW Group	CH Group	*P* value
Age (years)	46 (20)	45.5 (16.75)	49 (16.50)	0.480
BMI (kg/m^2^)	23.24 (2.92)	23.72 (2.4)	23.73 (3.47)	0.172
Gender (male/female, n)	8 / 29	8 / 28	9 / 28	0.959
ASA Physical status (I/II, n)	25 / 13	28 / 8	27 / 10	0.463
Anesthesia time (min)	130.16 ± 41.87	124.22 ± 41.53	121.03 ± 32.58	0.593
Surgery time (min)	107.5 ± 41.28	102 ± 39.64	100.68 ± 33.49	0.753
Basic MAP (mmHg)	91.67 (12.83)	89.83 (12.67)	93.67 (15.67)	0.165
Basic HR (bpm)	78.95 ± 11.82	79 ± 9.95	80.16 ± 10.03	0.761
Gastric volume (ml)	40.00 ± 17.31	37.21 ± 14.94	37.73 ± 16.08	0.734

Data are expressed as mean ± SDs, M (IQR) or number of patients (%) as appropriated

*CH group* Oral intake of 300 ml carbohydrate solution 2 h before surgery, *PW group* Oral intake of 300 ml pure water 2 h before surgery, *F group* Fasting for 8 h before surgery. *ASA* American Society of Anesthesiologists, *BMI* Body mass index. Basic MAP and HR: results of first measurement after admission

### Primary outcome

**Fig. 2:**
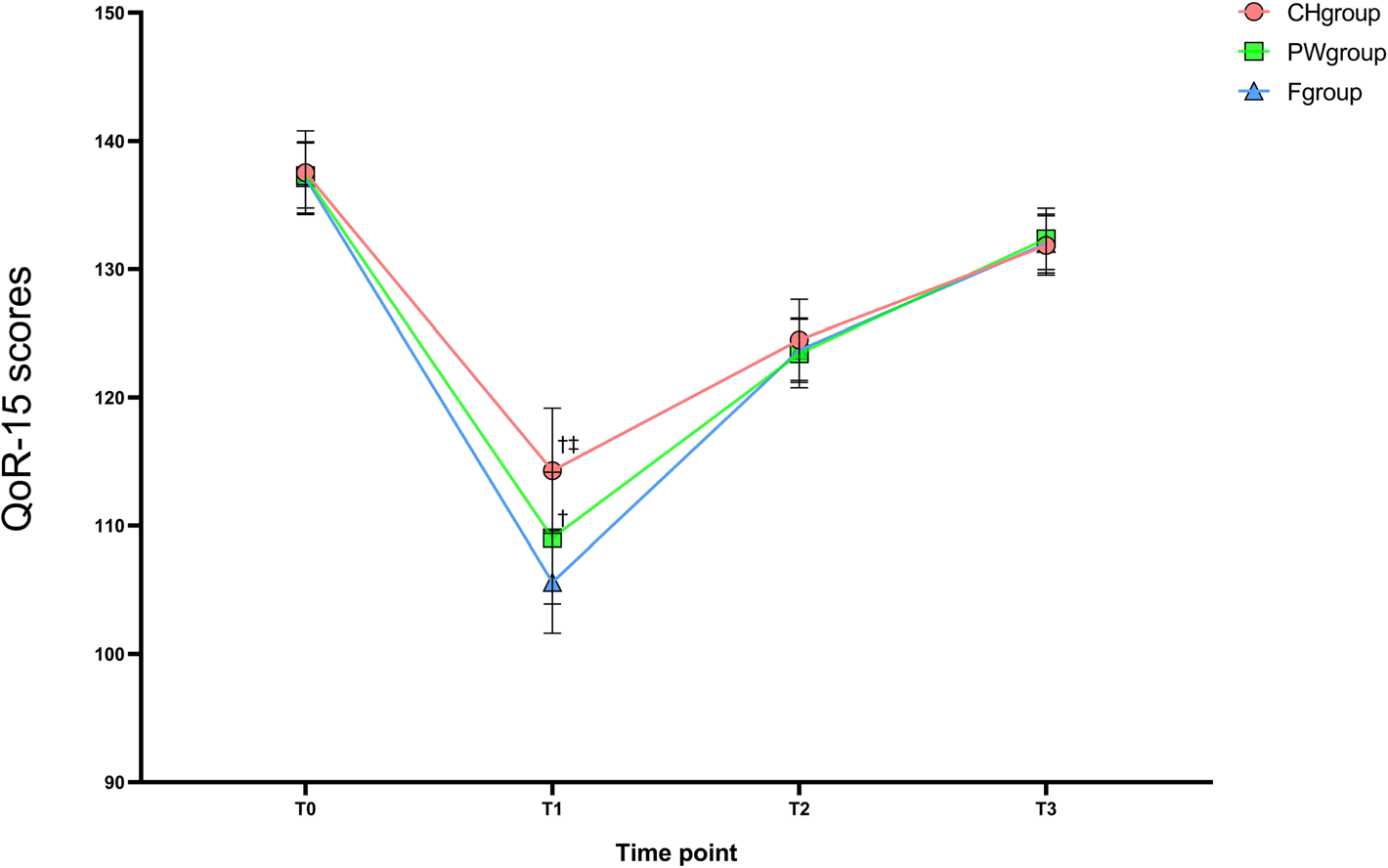
Total of QoR-15 scores varies over time among three groups. Data are presented as mean ± SDs or M (IQR). Details of the groups are shown in Table 1. T0: 1d before surgery; T1: 24h after surgery; T2: 48h after surgery; T3: 72h after surgery; QoR-15: Quality of Recovery-15 questionnaire. ^†^compared with F group the difference was significant at 0.05 level. ^‡^compared with PW group the difference was significant at 0.05 level

Preoperative QoR-15 scores had no significant difference among the three groups (*P* > 0.05). At T1, the total QoR-15 scores of the CH group and PW group were significantly greater than those in the F group (*P* < 0.05) and the total QoR-15 scores of the CH group were significantly greater than the PW group (*P* < 0.05). No significant difference was found among three groups at T2 and T3 (*P* > 0.05). Compared with T0, QoR-15 scores decreased significantly at other time points within groups (*P* < 0.05) (Fig. 2).

**Fig. 3:**
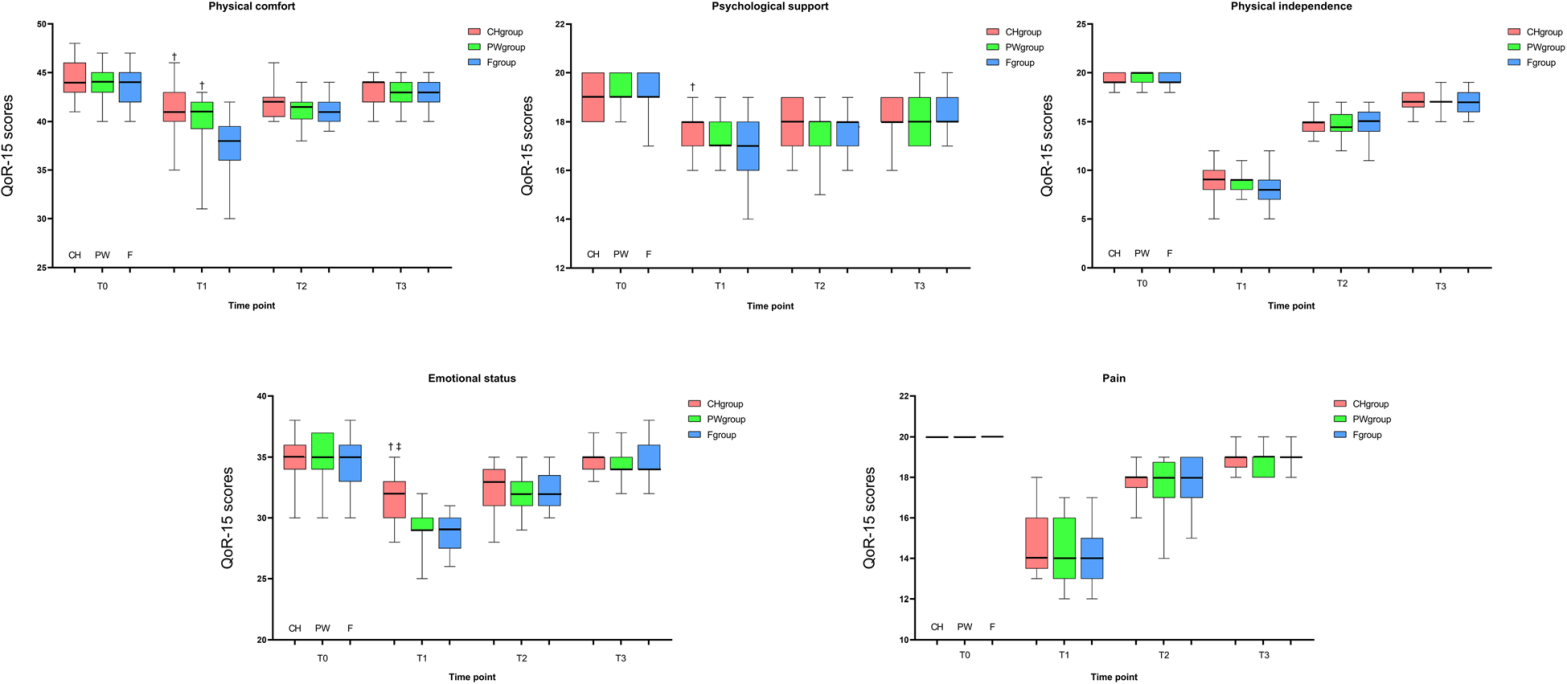
Each dimension varies over time among the three groups. ^†^compared with F group the difference was significant at 0.05 level. ^‡^compared with PW group the difference was significant at 0.05 level

Among the five dimensions of the QoR-15 at T1, scores of physical comfort (*P* < 0.05), psychological support (*P* < 0.05), and emotional dimension (*P* < 0.05) in the CH group were significantly higher compared to the F group; scores of physical comfort (*P* < 0.05) in the PW group was significantly higher than those in the F group; scores of emotional dimension (*P* < 0.05) in the CH group were significantly higher compared to the PW group. There was no difference in postoperative pain among the three groups (Fig. 3).

### Secondary outcomes

**Fig. 4:**
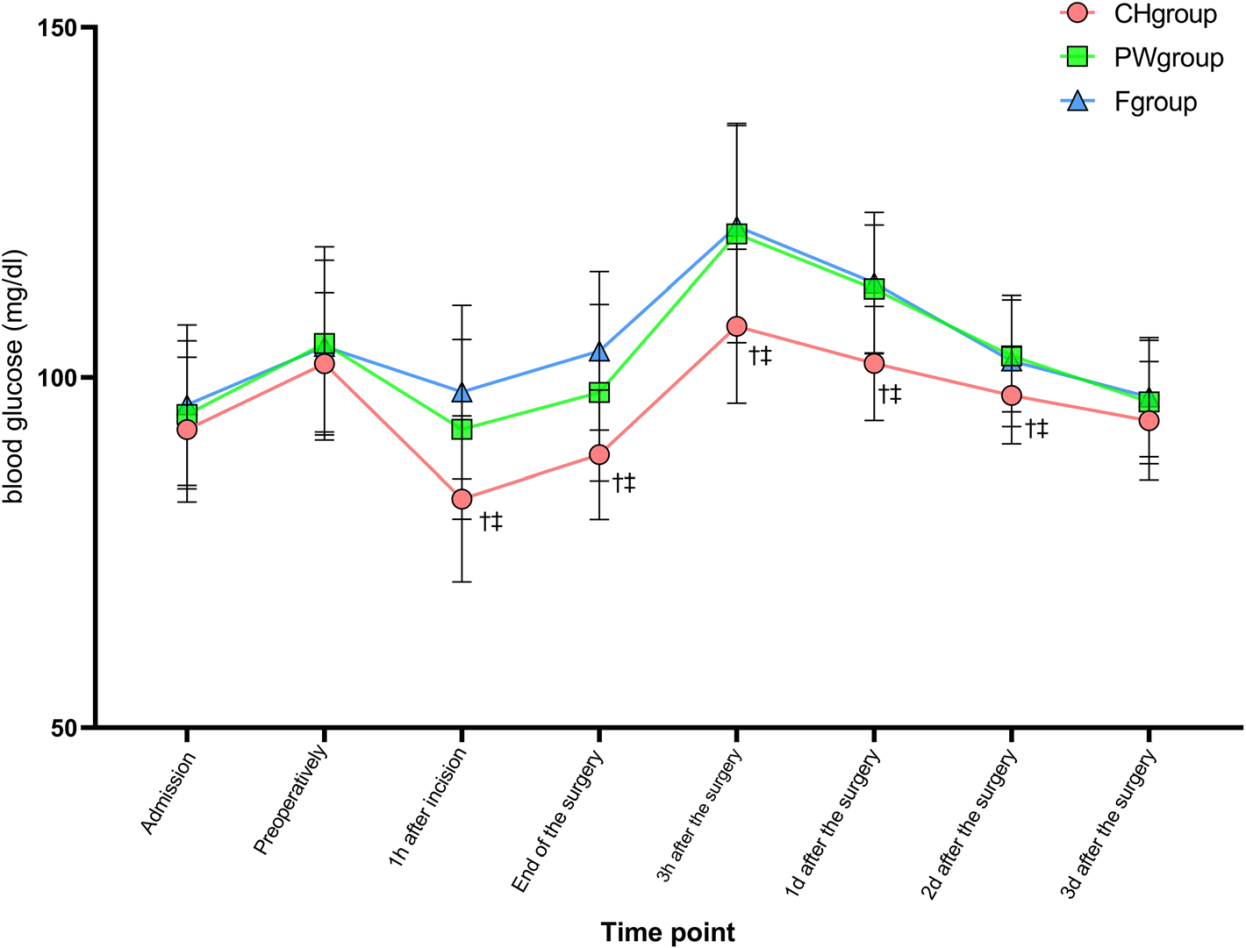
Blood glucose varies over time among three groups. ^†^compared with F group the difference was significant at 0.05 level. ^‡^compared with PW group the difference was significant at 0.05 level

No significant difference in blood glucose among the three groups of patients on admission and before surgery. Compared with the F and PW group, blood glucose in the CH group were significantly lower at 1 h after incision, end of the surgery, 3 h, 1d and 2d after the surgery, and there was no significant difference between the PW group and F group at each time point (Fig. 4).

The perioperative patient discomfort scores are shown in Table 2. Arrival in the operating theatre and after surgery, the CH group was significantly lower compared to the F group (*P* < 0.05). The incidence of ephedrine administration in the F group was significantly higher than that in the CH and PW group (*P* < 0.05). The minimum value of mean arterial pressure (MAP) in the CH group during induction was significantly higher compared to the F group (*P* < 0.05). The maximum value of heart rate (HR) in the CH group during intubation was significantly lower than the F group (*P* < 0.05) (Table 3).

**Table 2:** Comparison of patients’ scores in discomfort symptoms

	F Group	PW Group	CH Group	*P* value
Thirst				
Arrival in the operating theatre	5 (3)	4 (2)^a^	3 (3.5)^a^	0.000
3 h after surgery	8 (2.5)	6 (3)^a^	5 (3.5)^ab^	0.000
Hunger				
Arrival in the operating theatre	4 (4)	4.5 (2)	2 (3)^ab^	0.000
3 h after surgery	5 (2)	5 (2)	4 (2)^ab^	0.000
Anxiety				
1d before surgery	1 (0.5)	1 (1)	1 (1)	0.175
Arrival in the operating theatre	3 (1.5)	3 (1)^a^	3 (1)^a^	0.008
3 h after surgery	5 (1)	3 (1)^a^	3 (2)^a^	0.000
1d after surgery	3 (2)	3 (1.75)	2 (1)^ab^	0.000

Data are presented as M (IQR). Details of the groups are shown in Table 1

^a^compared with F group the difference was significant at 0.05 level

^b^compared with PW group the difference was significant at 0.05 level

**Table 3:** Intraoperative data comparisons among groups

	F Group	PW Group	CH Group	*P* value
Propofol (mg)				
Induction	120 (30)	130 (47.5)	130 (50)	0.144
Total	986 (463.5)	1005.5 (525.75)	988 (328.5)	0.677
Sufentanil (μg)				
Induction	20 (0)	20 (0)	20 (0)	0.294
Total	30 (0)	30 (0)	30 (0)	0.298
Cisatracurium				
Induction	8 (2.5)	8 (2)	9 (2)	0.166
Total	10 (3.5)	12 (2)	10 (4.5)	0.052
Midazolam (mg)	2 (0)	2 (0)	2 (0)	0.231
Remifentanil (μg)	478 (277.25)	552.5 (345.25)	460 (365)	0.719
Infusion (ml)	900 (412.5)	1000 (415)	930 (210)	0.141
Ephedrine (n)	23 (62.3%)	8 (22.2%)^a^	11 (29.7%)^a^	0.001
Atropine (n)	2 (5.4%)	2 (5.6%)	2 (5.4%)	0.999
Lowest MAP during induction (mmHg)	59.41 ± 5.10	61.62 ± 6.27	64.41 ± 6.95^a^	0.003
Lowest HR during induction (bpm)	65.11 ± 9.24	613.53 ± 8.61	61.70 ± 6.98	0.217
Highest MAP during intubation (mmHg)	98.90 ± 13.21	96.03 ± 15.42	92.64 ± 10.59	0.129
Highest HR during intubation (bpm)	87.43 ± 11.98	81.44 ± 11.85	75.54 ± 9.63^a^	0.000

Data are presented as mean ± SDs, M (IQR) or number of patients (%). Details of the groups are shown in Table 1

Induction: the period between the start of administration of anesthetic drugs and the end of the intubation; The liquid in our operation is the compound sodium chloride injection. Intubation: the period from the laryngoscopy enters the mouth to three minutes after the endotracheal tube is placed in the glottis

^a^compared with F group the difference was significant at 0.05 level

For the first postoperative anal exhaust time, we observed that the CH group and PW group had a significantly shorter time compared to the F group (*P* < 0.05); there was no significant difference between the CH group and PW group. No difference in anal first defecates time among the three groups (*P* > 0.05). The incidence of postoperative nausea in the CH and PW group was significantly lower than the F group (*P* < 0.05). The incidence of postoperative vomiting in the PW group was significantly lower than the F group (*P* < 0.05). No significant differences were observed among three groups on postoperative hospital stay and duration of drainage tube reservation (*P* > 0.05) (Table 4). Preoperative gastric volume had not significantly difference among groups (*P* > 0.05) (Table 5), no vomiting or aspiration occurred during intubation or extubation.

**Table 4:** Postoperative data comparisons among groups

	F Group	PW Group	CH Group	*P* value
Anal first exhaust time (hour)	19.78 ± 5.35	16.14 ± 4.68^a^	15.59 ± 5.10^a^	0.001
Anal first defecates time (hour)	39.24 ± 13.58	35.81 ± 9.52	31.84 ± 10.86^a^	0.024
Drainage tube extraction time (day)	3 (1)	3 (1)	3 (1)	0.781
Postoperative hospital stay (day)	4 (1)	4 (1.75)	3 (1.5)	0.977
Nausea (n)	13 (35.1%)	4 (11.1%)^a^	4 (10.8%)^a^	0.010
Vomiting (n)	10 (27.0%)	2 (5.6%)^a^	3 (8.1%)	0.014

Data are presented as mean ± SDs, M (IQR) or number of patients (%). Details of the groups are shown in Table 1

The time begins when the patient leaves the operating room. It is recorded as the first day after the operation from 0 a.m. on the night of the operation

^a^compared with F group the difference was significant at 0.05 level

**Table 5:** Comparison of preoperative gastric volume among groups

		F Group	PW Group	CH Group	*P* value
CSA (cm^2^)	Supine position	4.01 (0.90)	3.92 (0.77)	3.82 (0.84)	0.903
	Right lateral decubitus (RLD)	4.76 (0.56)	4.68 (0.80)	4.42 (0.85)	0.218
GV (ml)		40.00 ± 17.31	37.21 ± 14.94	37.73 ± 16.08	0.734

Data are presented as mean ± SDs, M (IQR). Details of the groups are shown in Table 1

CSA (cm^2^) = π × AP × CC/4, GV (ml) = 27.0 + 14.6 × right-lat CSA − 1.28 × age

*AP* The anteroposterior diameter of the gastric antrum, *CC* The craniocaudal diameter of the gastric antrum

## Discussion

This study examined the effect of preoperative oral low-concentration carbohydrate on patient-centered quality of postoperative recovery in patients undergoing thyroidectomy. We have found that even low-concentration carbohydrate can improve the postoperative recovery quality of patient self-evaluation and make the blood glucose more stable after surgery.

It has been determined that the minimal clinically important difference (MCID) for the QoR-15 is 8 points to conclude an effect exists [20, 21]. The mean value of QoR-15 scores in the CH group reached the MCID standard at T1 compared to the F group, rather than PW group. These results indicate that even low-concentration carbohydrate can also improve the quality of recovery at the patient aspect to 24 h after thyroidectomy with clinical significance. Preoperative oral intake of pure water can also statistically improve the QoR-15 scores at T1, however, its clinical benefits are limited. In our study, preoperative oral low-concentration carbohydrate can make patients feel relaxed, improve the sleep quality, and relieve the patient discomfort such as hunger, thirst and anxiety. In addition to the above advantages, it also increases patient comfort by reducing the incidence of postoperative nausea and vomiting, hyperglycemia and accelerating the gastrointestinal recovery. Based on the above advantages, preoperative oral low-concentration carbohydrate can improve the quality of postoperative recovery by improving the three dimensions of physical comfort, psychological support, and emotional status in QoR-15. Besides, our results also showed that the preoperative patient self-score and perioperative other outcomes of patients with low-concentration carbohydrate were both better than those of pure water or fasting group, so the patient-centered quality of postoperative recovery should be reliable.

The main objective of preoperative oral carbohydrate is to produce the change in metabolism that normally takes place when breakfast is eaten. This elicits an endogenous release of insulin that turns off the overnight fasting state of the metabolism [22]. Preoperative oral high-concentration carbohydrate can shorten the length of hospital stay on patients undergoing major operations by decreasing insulin resistance and improving postoperative recovery quality, such as colorectal surgery, coronary artery bypass graft surgery, but lacking of evidence about low-concentration carbohydrate [3, 23, 24]. And the degree of insulin resistance depends on the trauma and blood loss of surgeries [5, 25]. For minor operations with the low level of insulin resistance, preoperative oral high-concentration carbohydrate may be not suitable. Excessive carbohydrate will induce a large amount of insulin secretion, thereby inducing insulin resistance, which is not conducive to the blood glucose. A study shown that 2.5% of carbohydrate drinks could still improve postoperative insulin resistance [26]. Our results showed that the blood glucose in each group had a consistent change trend, the preoperative and postoperative blood glucose is higher than the basic value at admission, it may be related to the stress and insulin resistance. The postoperative blood glucose in the CH group was significantly lower than the PW group and F group, so it is possible to decrease insulin resistance in patients undergoing open thyroidectomy by taking low-concentration carbohydrate.

However, there was also a different result. Doo AR et al. [27] pointed out that preoperative oral high-concentration carbohydrate administration did not appear to improve patient well-being and satisfaction compared with midnight fasting in patients undergoing thyroidectomy. Compared to our study, they administered a higher concentration of carbohydrate solution, and the operation time and anesthesia time were shorter than ours. High-concentration carbohydrate may have little curative effect for quenching thirst, besides shorter operation and anesthesia time mean that fewer postoperative complications and discomfort because of more mild trauma and less consumption of anesthetic drugs. All of these mean that there was a high recovery quality of patients in their study. So, no significant difference between the two groups was observed in their trial. Compared with low-concentration carbohydrate, preoperative oral high-concentration carbohydrate may not be suitable for thyroidectomy. “Currently, high-concentration carbohydrate used in the clinic is more expensive than low-concentration carbohydrate. In addition, according to the patients’ feedback in the preliminary trials: the low-concentration carbohydrate we chose has better taste, lower price and more convenient availability than those high-concentration carbohydrates used clinically.”

There is no evidence to prove a positive effect on intraoperative heart rate and blood pressure by preoperative oral carbohydrate [28–30]. Our results showed that the incidence of ephedrine administration in the F group was significantly higher than the CH group and PW group. During the induction of anesthesia, the lowest mean arterial pressure (MAP) in the CH group was significantly higher compared to the F group, and the fastest heart rate during intubation in the CH group was significantly lower than the F group. Previous studies have not described the extreme values of intraoperative heart rate and blood pressure. Our result indicates that preoperative oral low-concentration carbohydrate has a positive effect on maintaining the stability of intraoperative heart rate and blood pressure. Patients with preoperative anxiety often associated with poor postoperative analgesia, prolonged hospital stay, high incidence of chronic pain, nausea and vomiting, but the mechanism of this phenomenon remains unclear [31, 32]. Although we found the improvement of perioperative anxiety by preoperative oral low-concentration carbohydrate, there was no significant difference in the Pain dimension among groups in this study. Surgery of our study had minor trauma to the patients, thus leading to an unobvious difference in pain scores. The postoperative recovery is based on a patient-centered approach that combines patient perceptions with objective perioperative outcomes. The comprehensive assessment model of patient-centered is consistent with the concept of comfortable medicine and Enhanced Recovery After Surgery (ERAS) [2] advocated by us and also provides direction for the future evaluation of postoperative recovery quality.

Gastric volume assessment by ultrasound helps to determine and avoid the risk of aspiration [13]. Although studies have confirmed the safety of oral intake of 200–400 ml carbohydrate solution 2 h before surgery [1, 14], a rigorous preoperative ultrasound gastric volume assessment was still performed to ensure patients’ safety in this study [13–19]. According to our results, no vomiting or aspiration occurred during intubation or extubation, no full stomach was observed, no significant difference of preoperative gastric volume was found among groups. Our results reconfirmed the safety of preoperative oral 300 ml low-concentration carbohydrate (4.8%) 2 h before surgery.

Unfortunately, if we measured the postoperative insulin resistance, we will have stronger evidence to show the effect of low-concentration carbohydrate on postoperative insulin resistance. Since we did not set a gradient of concentration for carbohydrate, our results did not reflect the optimal concentration of carbohydrate to improve the quality of postoperative recovery and decrease insulin resistance.

In summary, we proved that preoperative oral low-concentration carbohydrate could improve the quality of postoperative self-evaluation recovery and reduce the incidence of postoperative hyperglycemia on patients undergoing thyroidectomy. Routine administration of oral low-concentration carbohydrate to nondiabetic patients who are candidates for open thyroidectomy could reduce the risk of unidentified potentially dangerous hyperglycemia episodes in the vast majority of patients, but we still need more evidence to prove the effect of low-concentration carbohydrate on postoperative insulin resistance and postoperative recovery for minor surgeries.

## Data Availability

The datasets used during the current study are available from the corresponding author on reasonable request.

## Abbreviations

QoR-15: Quality of Recovery-15
BIS: Bispectral index
NRS: Numerical rating scale
PONV: Postoperative nausea and vomiting
ASA: American Society of Anesthesiologists
TCI: Target-controlled infusion
PACU: Post anesthesia care unit
BMI: Body mass index
ANOVA: One-way analysis of variance
SD: Standard deviation
IQR: Interquartile range
MAP: Mean arterial pressure
HR: Heart rate
ERAS: Enhanced Recovery After Surgery
